# Plasticity in Brainstem Mechanisms of Pain Modulation by Nicotinic Acetylcholine Receptors in the Rat

**DOI:** 10.1523/ENEURO.0364-16.2017

**Published:** 2017-02-01

**Authors:** Francis J. Jareczek, Stephanie R. White, Donna L. Hammond

**Affiliations:** 1 Medical Scientist Training Program, University of Iowa, Iowa City, IA 52242; 2 Interdisciplinary Graduate Program in Neuroscience, University of Iowa, Iowa City, IA 52242; 3 Department of Anesthesia, University of Iowa, Iowa City, IA 52242; 4 Department of Pharmacology, University of Iowa, Iowa City, IA 52242

**Keywords:** antinociception, complete Freund’s adjuvant, epibatidine, nicotinic acetylcholine receptors, rostral ventromedial medulla

## Abstract

Individuals with chronic pain may be driven to smoke more because the analgesic efficacy of nicotine diminishes. To determine whether persistent pain diminishes the actions of a nicotinic acetylcholine receptor (nAChR) agonist in pain modulatory pathways, we examined the effects of epibatidine in the rostral ventromedial medulla (RVM) of rats with and without inflammatory injury induced by intraplantar injection of complete Freund’s adjuvant (CFA). In uninjured rats, epibatidine produced a dose-dependent antinociception that was completely blocked by dihydro-β-erythroidine (DHβE; α4β2 antagonist) and partially blocked by methyllycaconitine (MLA; α7 antagonist). Epibatidine reversed heat hyperalgesia when microinjected in the RVM 4 h, 4 d, or 2 weeks after CFA treatment. Although DHβE completely blocked epibatidine’s antihyperalgesic effect at 4 h, at 2 weeks it elicited only partial antagonism. Methyllycaconitine was ineffective at both time points. Epibatidine’s antinociceptive efficacy in the uninjured hind paw progressively declined, and it was without effect 2 weeks after CFA. Moreover, as early as 4 h after CFA, the antinociceptive effect of epibatidine was no longer antagonized by DHβE. Neither antagonist alone altered paw withdrawal latency in uninjured or CFA-treated rats, suggesting that neither α4β2 nor α7 nAChRs are tonically active in the RVM. The *B*_max_ and *K_d_* of α4β2 nAChRs in the RVM were unchanged after CFA treatment. These observations provide the first evidence of pharmacological plasticity of the actions of α4β2 nAChR agonists in a critical brainstem pain modulatory pathway and may in part explain why people with chronic pain smoke more than the general population.

## Visual Abstract

**Figure:**
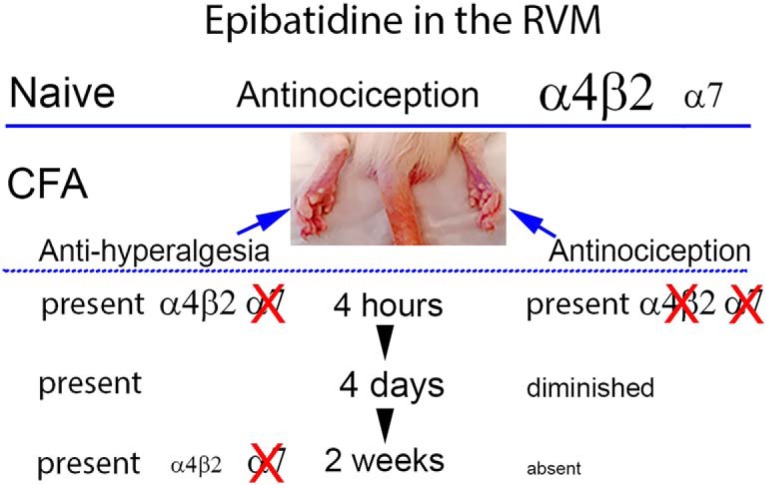

## Significance Statement

This article presents evidence that, over time, inflammatory injury diminishes the antinociceptive effects of an nAChR agonist in the brainstem and changes the receptors that mediate the agonist’s antihyperalgesic and antinociceptive effects. These data support the clinical hypothesis that those in chronic pain smoke more as a result of a decrease in the analgesic efficacy of nAChR activation.

## Introduction

Smoking and chronic pain both present significant health care burdens, and the well-established interaction between the two only worsens the overall toll they exert on both individuals and society (Centers for Disease Control and Prevention, 2004, 2010; Ditre et al., 2011; Institute of Medicine, 2011; World Health Organization, 2011). Observational studies find that smoking rates are positively correlated with pain severity and its resulting functional impairment, and analogously, smoking exacerbates both the intensity and associated impairment of chronic pain (Ditre and Brandon, 2008; Hooten et al., 2011; Patterson et al., 2012). Effectively, a positive feedback loop appears to be initiated: individuals smoke to relieve their pain, smoking exacerbates the pain, and individuals smoke more in response.

In nonsmoking humans, nicotine relieves acute pain and decreases opiate analgesic consumption in the acute postoperative setting (Hong et al., 2008; Yagoubian et al., 2011; Ditre et al., 2016). Nicotine has high affinity for α4β2 nicotinic acetylcholine receptors (nAChRs; Wonnacott and Barik, 2007; Wonnacott, 2014), and nAChR agonists that selectively target α4β2 nAChRs have shown efficacy in clinical trials but with a narrow therapeutic index (Decker et al., 2001; Jones and Dunlop, 2007; Rowbotham et al., 2009, 2012). Studies in rodents suggest that the spinal cord, periaqueductal gray (PAG), and rostral ventromedial medulla (RVM) are important sites of action of nAChR agonists (Umana et al., 2013). Of these, the RVM is of particular interest because it is a critical relay nucleus in endogenous pain modulatory pathways. The RVM receives afferents from more rostral nuclei (e.g., PAG, amygdala) implicated in modulating nociception (Heinricher et al., 2009; Ossipov et al., 2010) and in turn projects directly to the dorsal horn, where it either facilitates or inhibits the transmission of nociceptive information (Ren and Dubner, 1999, 2002; Millan, 2002; Vanegas, 2004; Vanegas and Schaible, 2004). Microinjection of the α4β2 nAChR agonist ABT-594 in the RVM is sufficient to produce antinociception (Bannon et al., 1998). Activation of α4β2 nAChRs specifically in the RVM also appears to be necessary for nicotinic antinociception: inactivation with lidocaine or microinjection of nAChR antagonists in the RVM antagonizes the antinociceptive effects of systemically administered nicotine and epibatidine (Curzon et al., 1998; Decker et al., 1998).

Many hypotheses have been put forward to explain why chronic pain is so poorly managed in smokers. The proposed mechanisms focus largely on biological processes such as inflammation, poor perfusion, ischemia, poor wound healing, nitric oxide production, and nutrition (Ditre et al., 2011). However, little attention has been paid to the role that adaptive changes in the CNS play in this phenomenon. Nociceptive sensitivity reflects the net sum of activity in bulbospinal pain facilitatory and pain inhibitory pathways. Persistent pain states effect substantial changes in the physiology and pharmacology of the bulbospinal pain pathways, altering the balance between facilitation and inhibition (Ren and Dubner, 1999, 2002; Vanegas, 2004; Vanegas and Schaible, 2004). These changes have ramifications for the antinociceptive potency and efficacy of drugs that act in key nuclei, such as enhancement of the antihyperalgesic and antinociceptive effects of µ-opioid receptor agonists in the RVM (Hurley and Hammond, 2001; Schepers et al., 2008).

We hypothesized that persistent pain states may lead to a time-dependent decrease in the antihyperalgesic and antinociceptive efficacy of nAChR agonists in the RVM. This hypothesis was tested by microinjection of epibatidine, a highly potent nAChR agonist, in the RVM of male rats at different times after induction of inflammatory injury by intraplantar injection of complete Freund’s adjuvant (CFA). The pharmacological specificity of epibatidine’s effects and possible tonic activation of nAChRs was further probed using antagonists of the α4β2 and α7 nAChR, which represent the two most abundant subtypes of nAChR in the CNS (Wu and Lukas, 2011; Hurst et al., 2013). As the most parsimonious explanation for a change in agonist action would be a loss of receptor number or decrease in affinity, we additionally conducted radioligand binding studies in rats with and without persistent inflammatory injury. The results of these studies partially support our hypotheses but also suggest alternate mechanisms. Taken together, our findings provide the first evidence of adaptive changes in a critical bulbospinal pathway through which nAChR agonists act to modulate nociception and support for the postulate that patients with chronic pain may smoke more because their ongoing pain decreases the analgesic effects of nicotine.

## Methods and Materials

### Animals

Adult (8–10 weeks of age) male Sprague-Dawley rats weighing 225–275 g were obtained from Charles River. Female rats were not used because the study was initiated before the National Institutes of Health mandated assessment of sex differences. Moreover, the phenotype of nicotine-induced analgesia is more consistent in men than in women (Shi et al., 2010). All experiments were approved by the University of Iowa Animal Care and Use Committee (protocol #4051048) and were conducted based on guidelines set forth by the National Institutes of Health and the International Association for the Study of Pain. After arrival, the animals were acclimated to the facility for at least 48 h before use. Animals were housed two per cage on eighth-inch corn cob bedding (cat. no. 7092; Envigo) in a temperature- and humidity-controlled room on a 12-h light/dark cycle with free access to food and water. After intracerebral cannula placement or intraplantar injection of CFA or saline, the rats were housed individually on the same bedding. Every effort was made to minimize the number of animals used and their suffering.

### Experimental design for behavioral studies

The first set of experiments was designed to assess the efficacy and pharmacological specificity of the highly potent nAChR agonist epibatidine microinjected in the RVM of naive rats and rats after different durations of inflammatory injury. Although the investigator could not be blinded to intraplantar treatment, the person testing the rats was blinded to the test drugs, which were made up by another person and assigned a letter that changed throughout the study. The experimental unit was the rat. Although sample size and power were not specifically determined before the study started, the numbers of rats are consistent with previous work from this laboratory.

### Model of hind paw inflammatory injury

A single injection of 150 µl CFA (150 μg *Mycobacterium butyricum*, 85% Drakeol 5NF, and 15% Aralacel A mannide monooleate emulsifier; cat. no. 344289; Calbiochem) was made into the plantar surface of the left hind paw of rats lightly anesthetized with isoflurane. Intraplantar injection of CFA induces a robust and reproducible inflammatory injury that begins within 1 h and persists for at least 2 weeks as assessed by paw edema and tests for heat and mechanical hyperalgesia (Nagakura et al., 2003; Hamity et al., 2010). The same volume of physiological saline (0.9%, pH 7.4) was injected in a separate group of animals as a control.

In the CFA model, the withdrawal threshold of the ipsilateral hind paw is greatly diminished, whereas that of the contralateral hind paw is unaffected. Therefore, one can assess the ability of agents to return the threshold of the ipsilateral hind paw to normal values (termed antihyperalgesia) as well as the ability of an agent to further increase the threshold of the contralateral hind paw beyond normal values (termed antinociception). As antihyperalgesia and antinociception are distinct phenomena, comparison of drug effects between ipsilateral and contralateral hind paws is inappropriate. The impact of chronic pain on the antinociceptive effects of drugs (i.e., ability to suppress acute physiological pain) can be assessed by comparison of effects on the contralateral hind paw of CFA-treated rats and naive rats, as well as to the contralateral hind paw of rats with different durations of persistent pain as in this study.

### Assessment of nociceptive threshold and paw thickness

Rats were acclimated to the testing environment for 30 min and then placed in individual Plexiglas chambers situated on a glass surface that was maintained at 25°C for another 30-min period of acclimation. Nociceptive sensitivity was measured using the paw withdrawal test, in which a high-intensity beam of light was focused on the plantar surface of the hind paw. The time required for the rat to lift its hind paw from the heat stimulus was termed the paw withdrawal latency (PWL). The high-intensity lamp was adjusted to elicit baseline paw withdrawal latencies of 8–12 s. If a withdrawal response did not occur within 20 s, the test was terminated to prevent tissue injury, and the rat was assigned this latency. Response latencies were determined for the left (injured) and right (uninjured) hind paws. Hind paw thickness in the dorsoventral axis was measured with digital calipers (Ted Pella) before and after intraplantar injection of saline or CFA. All testing was conducted in a small room designated for this work and was completed between the hours of 08:00 and 14:00.

### Microinjection studies

Rats were anesthetized with a mixture of ketamine (70 mg/kg i.p.), xylazine (10 mg/kg i.p.), and acepromazine (1 mg/kg i.p.) and implanted with an intracerebral guide cannula positioned above the RVM (26-gauge; Plastics One). Cannulae were affixed to the skull with stainless steel screws and dental acrylic, and a stainless steel stylet was inserted in the guide cannula to maintain patency. Cannulae were implanted 6 to 9 d before behavioral testing. The health status of the rats was monitored daily.

All agents were microinjected in a volume of 0.25 μl via a 33-gauge stainless steel injector needle that extended 3 mm beyond the guide cannula tip. Drug was microinjected over ∼10 s, and delivery was monitored by following the movement of an air bubble in the tubing that connected the injector to the syringe pump. The needle was left in place for 60 s after the injection to minimize diffusion of drug up the injection tract. Each rat was used once. Epibatidine (cat. no. E1145; Sigma-Aldrich), dihydro-β-erythroidine (DHβE; cat. no. 2349; Tocris), and methyllycaconitine (MLA; cat. no. M168; Sigma-Aldrich) were dissolved in saline. Saline at pH 7.4 was used as the control for all drugs. Doses of the antagonists were based on prior literature (Panagis et al., 2000; Laviolette and van der Kooy, 2003; Champtiaux et al., 2006; Tsutsui-Kimura et al., 2010). After behavioral testing, animals were killed by CO_2_ inhalation. Brains were removed, fixed in 10% formalin containing 30% sucrose, and transverse sections cut on a cryostat microtome and stained with Cresyl Violet. Injection sites were identified by two individuals blinded to the behavioral outcome. Microinjection sites that were located in the nucleus reticularis gigantocellularis pars alpha were included with those in the nucleus raphe magnus and collectively referred to as the RVM. Rats were excluded if there was substantial tissue damage at the microinjection site.

### Experimental design for radioligand binding studies

Radioligand binding was used to determine and compare the *B*_max_ and *K_d_* of α4β2 nAChRs in the RVM 4 h, 4 d, and 2 weeks after intraplantar injection of saline or CFA. An experiment consisted of a cohort of saline- and CFA-treated rats at each of the three time points. Tissue from three rats was pooled to generate a sample, which served as the experimental unit. Three to five independent experiments (biological replicates) were conducted. Although sample size and power were not determined before the study started, the number of replicates is in line with previous work from this laboratory. The individual conducting the binding was blinded to the treatment condition of the homogenates. Rats used for radioligand binding were not subjected to thermal stimuli, as this nociceptive testing has been demonstrated to release endogenous substance P in the RVM (Hamity et al., 2014). In the case of acetylcholine, such release could interfere with determination of *B*_max_ and *K_d_*. However, paw thickness was measured to confirm the presence of inflammation induced by CFA.

### Tissue collection for radioligand binding

Four hours, 4 d, or 2 weeks after intraplantar saline or CFA injection, paw thicknesses were reassessed. The animals were then killed with CO_2_, exsanguinated, and decapitated. The brainstem was rapidly dissected out, and two transverse cuts were made 3 and 5 mm rostral to the obex. The resulting 2-mm-thick section was placed on a chilled surface and a triangular region defined at the vertex by the fourth ventricle and at each corner of the lateral edge of the pyramids was dissected free. The pyramids were then removed. The resulting region contained the nucleus raphe magnus and the adjacent bilateral nucleus reticularis gigantocellularis pars alpha. The freshly dissected tissue was collected into Eppendorf tubes on ice and stored at –80°C for no more than 2 d before being used to prepare membrane homogenates for radioligand binding.

### Membrane homogenate preparation

RVM triangles from three rats in each experimental group were combined, weighed, and thoroughly homogenized in 20 volumes of ice-cold assay buffer (50 mm TRIZMA, pH 7.4; Sigma-Aldrich). The homogenate was then centrifuged at 39,000 × *g* at 4°C for 15 min. The supernatant was discarded, 20 volumes of ice cold assay buffer were added, and the pellet was homogenized. The homogenate was incubated at 25°C for 10 min before a second centrifugation step at 39,000 × *g* at 4°C for 15 min. The supernatant was again discarded, and the pellet was resuspended in assay buffer. Protein concentration was determined using the Lowry assay. The membrane preparation was stored at –80°C until used for radioligand binding. Membrane preparations were coded to blind the investigator to treatment condition.

### Radioligand binding in membrane homogenates

Dilutions of stock [^3^H]epibatidine (62.2 Ci/mmol, lot #1730657 or 54.1 Ci/mmol, lot #1778835 or #1828391; cat. no. NET1102; Perkin Elmer) were prepared in assay buffer (50 mm TRIZMA, pH 7.4). All reactions were performed in triplicate with 50 µg membrane protein. Binding reactions were terminated by rapid filtration using a Brandel cell harvester and filter paper (Brandel #FP-100 GF/B) that had been soaked in 50 mm TRIZMA, pH 7.2, with 0.5% polyethyleneimine (w/v) at 4°C for at least 2 h to minimize nonspecific binding.

#### Kinetic assay: association

Test tubes were prepared with 50 µg of membrane protein and final concentrations of 0.002, 0.02, or 0.2 nm [^3^H]epibatidine. The membranes and radioligand incubated at 25°C in a shaking water bath for 0.5–5 h. Nonspecific binding was determined in the presence of 300 μm nicotine (cat. no. N5260; Sigma-Aldrich). Filter discs were washed three times with 5 ml of ice-cold wash buffer (50 mm TRIZMA, pH 7.2), and then placed in scintillation vials to which 500 μl of absolute ethanol was added. Five milliliters of scintillation fluid (Econo-Safe; RPI Corporation) was added 15 min later, and the vials were shaken for 15 min. Samples were left undisturbed for at least 15 h, then were agitated once more and counted using a Beckman Coulter LS6500 scintillation counter at 40% efficiency.

#### Kinetic assay: dissociation

Test tubes were prepared as described above with a concentration of 0.02 nm [^3^H]epibatidine. Samples were incubated at 25°C in a shaking water bath for 3 h to allow the binding reaction to reach equilibrium. Dissociation of [^3^H]epibatidine was then initiated by addition of 300 μm nicotine. The samples were then incubated for an additional 1–11 h. Nonspecific binding was determined at the 3- and 11-h time points in the presence of 300 μm nicotine for the duration of the experiment. The reaction was terminated, and samples were prepared for scintillation analysis as described above.

#### Saturation assay

Test tubes were prepared as described above with final [^3^H]epibatidine concentrations ranging from of 0.001 to 2.5 nm. Samples were incubated at 25°C in a shaking water bath for 3 h to allow the binding reaction to reach equilibrium. Nonspecific binding was determined in the presence of 300 μm nicotine. The reaction was terminated, and samples were prepared for scintillation analysis as described above. Aliquots of the different [^3^H]epibatidine dilutions were prepared for scintillation analysis to generate concentration-versus-CPM curves.

### Statistical analysis

#### Microinjection studies

Data were expressed as the mean ± SEM. Two-way repeated-measures ANOVA, in which the repeated factor was time and the other factor was treatment, was used to compare PWL among the different groups. Significant overall effects of treatment or time, or a significant interaction of treatment and time, were followed by the Holm–Sidak test for *post hoc* comparisons among mean values for the individual treatment groups. Only *p* values are reported for *post hoc* tests. Welch’s corrected *t*-test was used to compare paw thickness at each time point because it does not assume both groups have equal variance. Statistical analyses were conducted with SigmaPlot 13.0, which also indicated that assumptions of normality (Shapiro–Wilk test) and equal variance (Brown–Forsyth test) were met. A *p* < 0.05 was considered significant for this and all subsequent tests.

#### Radioligand binding studies

Saturation binding curves of specific binding were fitted by nonlinear regression using GraphPad Prism, and an *F*-test was conducted to determine whether the curves were best fit by a one- or two-site model. If the data were better fitted by a two-site model, the *B*_max_ and *K_d_* for the high-affinity site were used for analysis. The *B*_max_ and *K_d_* values were determined for each of three to five independent experiments and were averaged to generate the mean and SEM. Two-way ANOVA in which treatment was one factor and time was the other factor was used to compare *B*_max_ and *K_d_* values in saline- and CFA-treated rats at each time point. Superscript letters listed with *p*-values correspond to the statistical tests shown in Table 1.

**Table 1.** Statistical analysis.

Line	Data structure	Type of test	Power
a	Normal distribution	Two-way repeated-measures ANOVA (repeated factor: time; other factor: treatment) followed by Holm–Sidak test	For treatment: 0.944 For time: 1.000 For interaction: 0.669
b	Normal distribution	One-way repeated-measures ANOVA (repeated factor: time)	Too small to calculate
c	Normal distribution	Two-way repeated-measures ANOVA (repeated factor: time; other factor: treatment) followed by Holm–Sidak test	For treatment: 0.458 For time: 1.000 For interaction: 0.598
d	Normal distribution	Two-way repeated-measures ANOVA (repeated factor: time; other factor: treatment) followed by Holm–Sidak test	For treatment: 0.0734 For time: 0.999 For interaction: 0.349
e	Normal distribution	Welch’s *t*-test	Four hour 95% CI: 2.7–3.5 mm Four day 95% CI: 3.2–3.9 mm Two week 95% CI: 3.4–4.5 mm
f	Normal distribution	Two-way repeated-measures ANOVA (repeated factor: time; other factor: treatment) followed by Holm–Sidak test	For treatment: 0.998 For time: 1.000 For interaction: 0.980
g	Normal distribution	Two-way repeated-measures ANOVA (repeated factor: time; other factor: treatment) followed by Holm–Sidak test	For treatment: 0.856 For time: 1.000 For interaction: 0.959
h	Normal distribution	Two-way repeated-measures ANOVA (repeated factor: time; other factor: treatment) followed by Holm–Sidak test	For treatment: 0.846 For time: 1.000 For interaction: 0.691
i	Normal distribution	Two-way repeated-measures ANOVA (repeated factor: time; other factor: treatment) followed by Holm-Sidak test	For treatment: 0.725 For time: 1.000 For interaction: 0.880
j	Normal distribution	Two-way repeated-measures ANOVA (repeated factor: time; other factor: treatment) followed by Holm–Sidak test	For treatment: 0.202 For time: 1.000 For interaction: 0.501
k	Normal distribution	Two-way repeated-measures ANOVA (repeated factor: time; other factor: treatment) followed by Holm–Sidak test	For treatment: 0.115 For time: 0.961 For interaction: Too small to calculate
l	Normal distribution	Two-way repeated-measures ANOVA (repeated factor: time; other factor: treatment) followed by Holm–Sidak test	For treatment: 0.804 For time: 1.000 For interaction: 0.250
m	Normal distribution	Two-way repeated-measures ANOVA (repeated factor: time; other factor: treatment) followed by Holm–Sidak test	For treatment: 0.250 For time: 1.000 For interaction: 0.363
*n*	Normal distribution	Two-way repeated-measures ANOVA (repeated factor: time; other factor: treatment) followed by Holm–Sidak test	For treatment: 0.456 For time: 1.000 For interaction: 0.129
o	Normal distribution	Two-way repeated-measures ANOVA (repeated factor: time; other factor: treatment) followed by Holm–Sidak test	For treatment: 0.318 For time: 0.892 For interaction: 0.194
p	Normal distribution	Two-way repeated-measures ANOVA (repeated factor: time; other factor: treatment) followed by Holm–Sidak test	For treatment: too small to calculate For time: 1.000 For interaction: too small to calculate
q	Normal distribution	Two-way repeated-measures ANOVA (repeated factor: time; other factor: treatment) followed by Holm–Sidak test	For treatment: too small to calculate For time: 0.730 For interaction: too small to calculate
r	Normal distribution	Two-way ANOVA (factors: time, treatment)	For treatment: 0.050 For time: 0.195 For interaction: 0.050
s	Normal distribution	Two-way ANOVA (factors: time, treatment)	For treatment: 0.050 For time: 0.050 For interaction: 0.050
t	Normal distribution	Welch’s *t*-test	Four hour 95% CI: 3.9–4.2 mm Four day 95% CI: 3.4–4.4 mm Two week 95% CI: 3.1–3.8 mm

CI, confidence interval.

## Results

### Actions of epibatidine in the RVM of naive rats

**Fig. 1.**
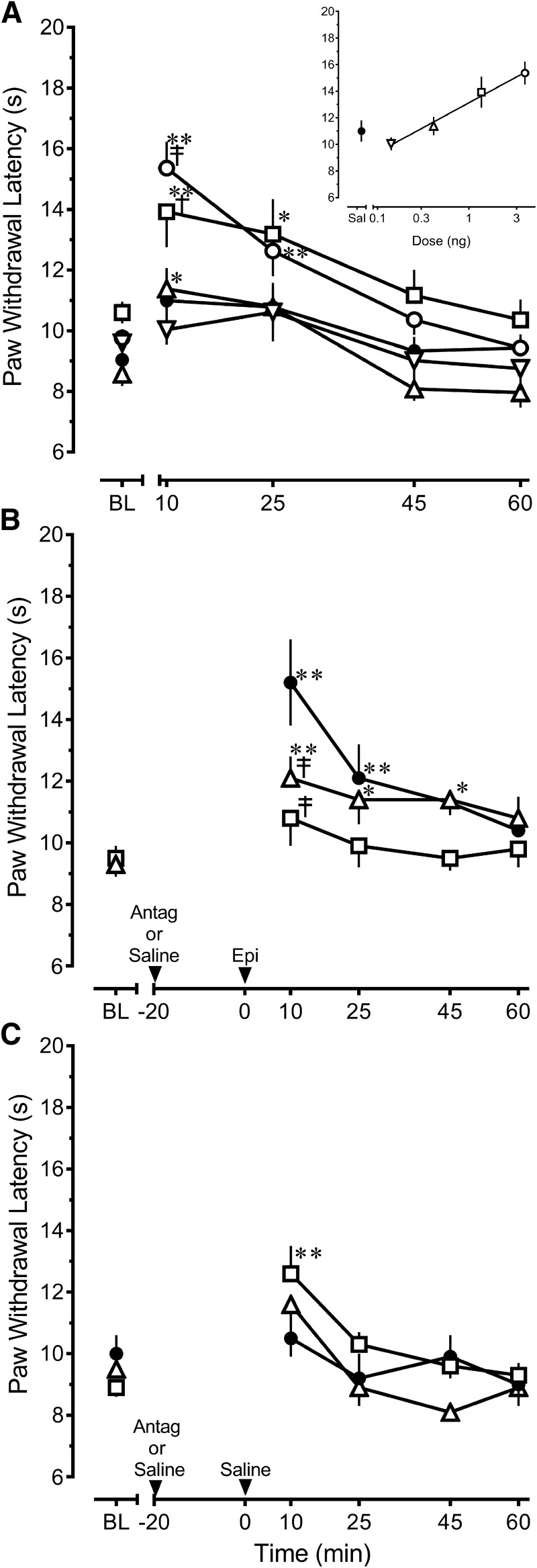
The antinociception produced by epibatidine in the rostral ventromedial medulla of uninjured rats is principally mediated by α4β2 nAChRs, which do not appear to be tonically active. ***A***, Microinjection of epibatidine (Epi) produces a dose-dependent increase in PWL to noxious heat ○: 4.11 ng, *n* = 14; ☐: 1.37 ng, *n* = 6; ▵: 0.41 ng, *n* = 5; ▿: 0.14 ng, *n* = 5; ●: saline, *n* = 9. ***B***, Prior microinjection of 5 µg of the α4β2 nAChR antagonist DHβE ▫, *n* = 6) completely blocks the effect of the highest dose of epibatidine (4.11 ng) compared to pretreatment with saline (●, *n* = 6), whereas 1 µg MLA (▵, *n* = 7) produces a partial antagonism. ***C***, Compared with saline (●, *n* = 6), microinjection of 5 µg DHβE (▫, *n* = 8) or 1 µg MLA (▵, *n* = 5) did not alter PWL in rats that subsequently received saline at the same site. Data are mean ± SEM. Latencies for left and right hind paws were averaged to yield a single value for each rat in all panels. BL, baseline PWL. *p < 0.05, **p < 0.01 compared with baseline values. ^†^*p* < 0.05, ^‡^*p* < 0.01 compared with saline control at the corresponding time point. Two-way repeated-measures ANOVA followed by Holm–Sidak test.

**Fig. 2.**
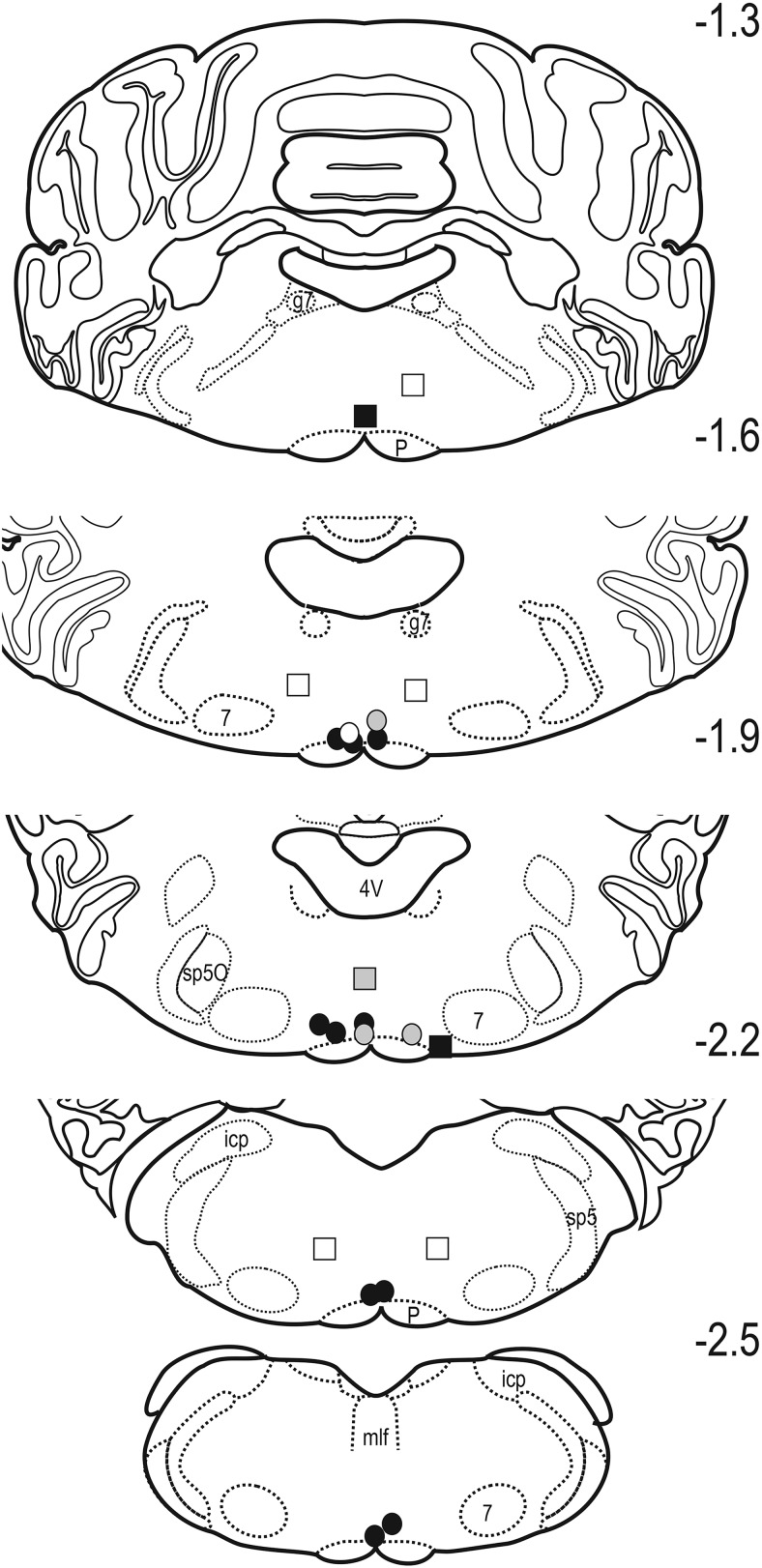
Distribution of sites in the RVM of uninjured rats at which 4.11 ng epibatidine was microinjected. The symbols encode the magnitude of the effect determined 10 min after microinjection. Gray and black circles, respectively, indicate that PWL was at least 2 and 3 SD greater than the mean baseline latency for the treatment group. Sites outside the RVM are indicated by similarly coded squares. Sites at which PWL changed by <1 SD are indicated by open symbols. 4V, fourth ventricle; g7, genu of the facial nerve; icp, inferior cerebellar peduncle; mlf, medial longitudinal fasciculus; P, pyramid; sp5O, spinal trigeminal nucleus pars oralis; sp5, spinal trigeminal tract; 7, facial motor nucleus. Numbers indicate distance in millimeters from the interaural line.

Microinjection of epibatidine throughout the rostral-caudal extent of the RVM of naive rats produced a dose- and time-dependent increase in PWL (treatment *F*_(4,34)_ = 6.208, *p* < 0.001; time *F*_(4,136)_ = 22.363, *p* < 0.001; interaction *F*_(16,136)_ = 2.040, *p* =0.015)^a^ (Fig. 1*A* and inset). The increase was short-lived, with peak effect observed within 10 min of injection (*p* < 0.001). Fig. 2 depicts the distribution of microinjection sites in the RVM for the 4.11-ng treatment group, which is representative of the distribution of RVM sites in all other treatment groups. In general, microinjection of the highest dose of epibatidine at sites dorsal to or outside the RVM did not increase PWL compared with baseline values (10.9 ± 0.8 s at 10 min; *n* = 8; one-way ANOVA *F*_(7,28)_ = 0.818, *p* = 0.525)^b^, although sites that impinged on the lateral or rostral borders of the RVM could yield modest increases in PWL.

The antinociceptive effect of epibatidine was challenged by pretreatment with 5 µg DHβE, a competitive α4β2 nAChR antagonist (Daly, 2005; Wonnacott and Barik, 2007; Wonnacott, 2014) or 1 µg MLA, a competitive α7 nAChR antagonist (Daly, 2005; Wonnacott and Barik, 2007; Wonnacott, 2014). These two antagonists differentially attenuated the effects of epibatidine (treatment *F*_(2,16)_ = 3.696, *p* = 0.048; time *F*_(4,62)_ = 11.328, *p* < 0.001; interaction *F*_(8,62)_ = 2.439, *p* = 0.023)^c^. Pretreatment with DHβE completely blocked the antinociceptive effect of the highest dose of epibatidine (*p* < 0.001), whereas challenge with MLA partially attenuated the antinociceptive effects of epibatidine (*p* = 0.003; Fig. 1*B*). One rat in the latter group adopted a cataleptic stance 45 min after injection, which interfered with PWL measurements at 45 and 60 min. A higher dose of MLA, 3 µg, by itself significantly increased PWL (data not shown), which precluded challenge of epibatidine with a higher dose of MLA. These data suggest an additional involvement of α7 nAChRs in the effects of the highest dose of epibatidine.

To assess whether tonic activation of nAChRs was present, 5 µg DHβE or 1 µg MLA was injected 20 min before microinjection of saline in the RVM. This design mirrored the design used in the pharmacological challenge of epibatidine so that the effect of each antagonist was assessed at the same time point as when its antagonism of epibatidine was determined. Compared with the effect of saline, microinjection of either 5 µg DHβE or 1 µg MLA did not alter PWL (treatment *F*_(2,16)_ = 1.181, *p =* 0.332; interaction *F*_(8,64)_ = 1.824, *p* = 0.089)^d^, suggesting that little tonic activation of α4β2 or α7 nAChRs exists in the RVM (Fig. 1*C*). Within-treatment comparison (time *F*_(4,64)_ = 10.012, *p* < 0.001) indicated that microinjection of 5 µg DHβE transiently increased paw withdrawal compared with baseline (*p* < 0.001), whereas saline did not (*p* = 0.923). Nonetheless, the lack of treatment effect does not support tonic activation of α4β2 nAChRs in the RVM.

### Impact of peripheral inflammatory injury on the antihyperalgesic and antinociceptive actions of epibatidine in the RVM

Intraplantar injection of CFA in the hind paw uniformly produced robust and reproducible inflammation as indicated by hind paw edema at 4 h, 4 d, and 2 weeks after injection. The thickness of the CFA-injected hind paw was significantly greater than baseline thickness at each time point (4 h: 10.3 ± 0.1 vs. 6.3 ± 0.01 (*n* = 38); 4 d: 9.9 ± 0.2 vs. 6.0 ± 0.05 (*n* = 13); 2 weeks: 9.6 ± 0.2 vs. 6.2 ± 0.01 mm (*n* = 60; 4 h Welch-corrected *t* = 69.02, df = 40.16; 4 d Welch-corrected *t* = 17.02, df = 13.09; 2 weeks Welch-corrected *t* = 21.22, df = 60.11, p < 0.001 for all three time points) or the uninflamed, contralateral hind paws (data not shown).

**Fig. 3.**
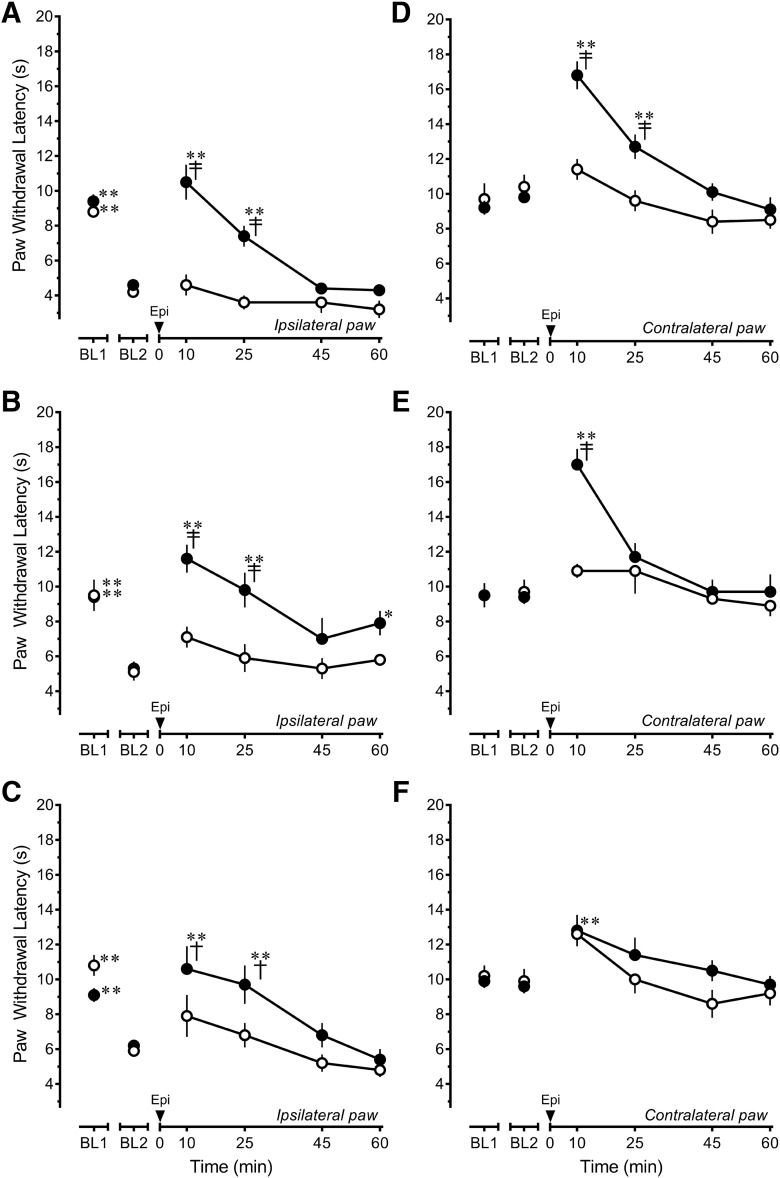
The antihyperalgesic effect of epibatidine persists, whereas its antinociceptive effect declines in a time-dependent manner after peripheral inflammatory injury. Epibatidine (Epi; 4.11 ng, ●) or saline (○) was microinjected in the rostral ventromedial medulla of rats 4 h (***A***, ***D***; saline *n* = 4, epibatidine *n* = 10), 4 d (***B***, ***E***; saline *n* = 5, epibatidine *n* = 8), or 2 weeks (***C***, ***F***; saline *n* = 6, epibatidine *n* = 11) after intraplantar injection of CFA in the left hind paw. ***A–C***, Ipsilateral, inflamed hind paw. ***D–F***, Contralateral, uninflamed hind paw. Data are mean ± SEM. BL1 refers to paw withdrawal latency before and BL2 refers to paw withdrawal latency after injection of CFA. *p < 0.05, **p < 0.01 compared with BL2 values. ^†^*p* < 0.05, ^‡^*p* < 0.01 compared with saline control at the corresponding time point. Two-way repeated-measures ANOVA followed by Holm–Sidak test.

Epibatidine completely reversed heat hyperalgesia within 10 min of its microinjection in the RVM. The effect dissipated over the next 45 min. Of note, epibatidine did not further increase PWL beyond baseline values. The antihyperalgesic effect of epibatidine was unchanged 4 h (treatment *F*_(1,12)_ = 28.466, *p* < 0.001; time *F*_(5,60)_ = 22.568, *p* < 0.001; interaction *F*_(5,60)_ = 6.229, *p* < 0.001)^f^, 4 d (treatment *F*_(1,11)_ = 11.726, *p* = 0.006; time *F*_(5,54)_ = 11.108, *p* < 0.001; interaction *F*_(5,54)_ = 3.272, *p* = 0.012)^g^, and 2 weeks (treatment *F*_(1,15)_ = 2.431, *p* = 0.140; time *F*_(5,75)_ = 13.682, *p* < 0.001; interaction *F*_(5,75)_ = 2.507, *p* = 0.037)^h^ after CFA treatment (Fig. 3*A–C*). In contrast, the antinociceptive effect of epibatidine in the contralateral, uninflamed hind paw progressively decreased in a time-dependent manner in CFA-treated rats (Fig. 3*D–F*). As in naive rats, microinjection of 4.11 ng epibatidine in the RVM of rats treated 4 h earlier with CFA significantly increased PWL of the uninjured hind paw for a period of 25 min, with the peak effect at 10 min (treatment *F*_(1,12)_ = 11.865, *p* = 0.005; time *F*_(5,60)_ = 15.555, *p* < 0.001; interaction *F*_(5,60)_ = 5.526, *p* < 0.001)^i^ (Fig. 3*D*). When examined 4 d after CFA (Fig. 3*E*), the magnitude of the increase in PWL was unchanged, but the duration of the effect was truncated (treatment *F*_(1,11)_ = 8.903, *p* = 0.013; time *F*_(5,54)_ = 10.662, *p* < 0.001; interaction *F*_(5,54)_ = 4.445, *p* = 0.002)^j^. In rats that had received an injection of CFA 2 weeks earlier, microinjection of 4.11 ng epibatidine in the RVM did not increase PWL in the contralateral hind paw compared to the effects of saline (treatment *F*_(1,15)_ = 1.625, *p* = 0.222; time *F*_(5,75)_ = 5.505, *p* < 0.001; interaction *F*_(5,75)_ = 0.715, *p* = 0.614)^k^.

**Fig. 4.**
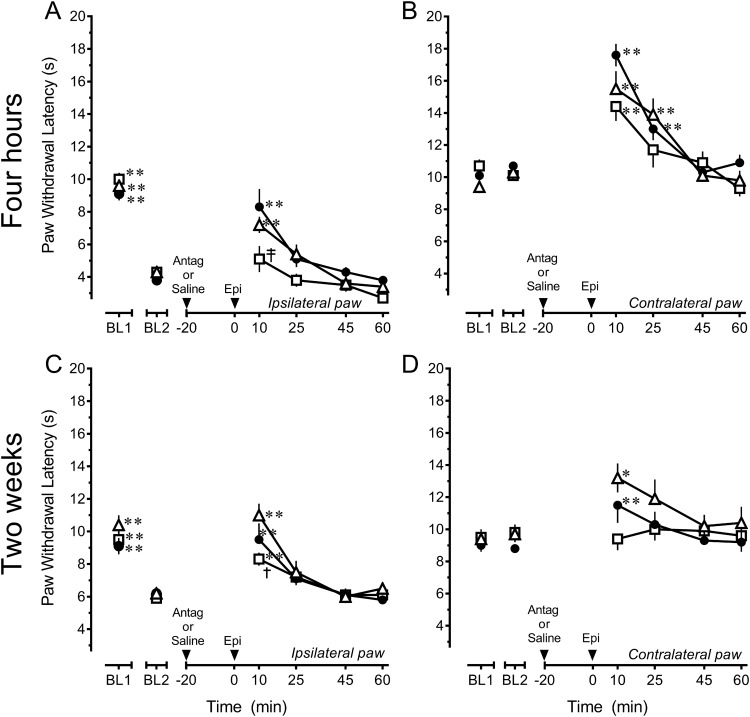
Persistent inflammatory injury alters the mechanisms by which epibatidine modulates nociception in the rostral ventromedial medulla in a time-dependent manner. ***A***, Microinjection of 5 μg DHβE (☐, *n* = 8) completely antagonizes while 1 μg MLA (▵, *n* = 8) does not diminish the antihyperalgesic effect of 4.11 ng epibatidine (Epi) in rats that received an intraplantar injection of CFA 4 h earlier compared to saline (●, *n* = 9). ***B***, Microinjection of 5 μg DHβE (☐, *n* = 8) or 1 μg MLA (▵, *n* = 8) did not antagonize the increase in paw withdrawal latency of the contralateral hind paw in rats that received an intraplantar injection of CFA 4 h earlier compared to saline (●, *n* = 9). ***C***, Microinjection of 5 μg DHβE (☐, *n* = 9) only partially antagonizes while 1 μg MLA (▵, *n* = 5) does not diminish the antihyperalgesic effect of 4.11 ng epibatidine in rats that received an intraplantar injection of CFA 2 weeks earlier compared to saline (●, *n* = 9). ***D***, Microinjection of 5 μg DHβE (☐, *n* = 9) or 1 μg MLA (▵, *n* = 5) before 4.11 ng epibatidine did not alter PWL of the contralateral hind paw in rats that received an intraplantar injection of CFA 2 weeks earlier compared to saline before 4.11 ng epibatidine (●, *n* = 9). Data are mean ± SEM. BL1 refers to paw withdrawal latency before and BL2 refers to paw withdrawal latency after injection of CFA. *p < 0.05, **p < 0.01 compared with BL2 values. ^†^*p* < 0.05, ^‡^*p* < 0.01 compared with saline control at the corresponding time point. Two-way repeated-measures ANOVA followed by Holm–Sidak test.

Given that the antihyperalgesic and antinociceptive effects of epibatidine were differently affected in the presence of persistent inflammatory injury, the pharmacological specificity of epibatidine was reexamined both 4 h and 2 weeks after CFA treatment. Four hours after CFA, comparison of the effects of DHβE or MLA to that of saline for the ipsilateral inflamed hind paw (treatment *F*_(2,22)_ = 6.596, *p* = 0.006; time *F*_(5,110)_ = 81.184, *p* < 0.001; interaction *F*_(10,110)_ = 1.505, *p* = 0.147)^l^ indicated that challenge with 5 µg DHβE completely blocked the antihyperalgesic effect of epibatidine (*p* = 0.003; Fig. 4*A*). Challenge with 1 μg MLA did not attenuate the antihyperalgesic effects of epibatidine at this time point (*p* = 0.287). Surprisingly, analysis of the contralateral uninflamed hind paw (treatment *F*_(2,22)_ = 2.291, *p* = 0.125; time *F*_(5,110)_ = 37.910, *p* < 0.001; interaction *F*_(10,110)_ = 1.728, *p* = 0.083)^m^ indicated that neither DHβE (*p* = 0.101) nor MLA (*p* = 0.151) attenuated the antinociceptive effects of epibatidine (Fig. 4*B*), unlike findings in naive rats (Fig. 1*B*). These data indicate that the antihyperalgesic effects in the period immediate to injury were predominantly mediated by α4β2 nAChRs, but that the antinociceptive effects of epibatidine were no longer mediated by either α4β2 or α7 nAChRs.

Given that the findings at 4 h suggested alterations in the pharmacology of epibatidine, additional studies were conducted 2 weeks after CFA treatment. At this time (treatment *F*_(2,20)_ = 3.584, *p* = 0.047; time *F*_(5,100)_ = 38.528, *p* < 0.001; interaction *F*_(10,100)_ = 1.238, *p* =0.276)*^n^*, challenge with 5 µg DHβE only partially antagonized the antihyperalgesic effects of epibatidine at 10 min (*p* = 0.044 at 10-min time point), and challenge with 1 μg MLA was ineffective (*p* = 0.083; Fig. 4*C*). These findings suggest that the antihyperalgesic effect was not substantially mediated by α4β2 or α7 nAChRs. In the presence of DHβE or MLA, epibatidine continued to lack antinociceptive efficacy in the contralateral hind paw (treatment *F*_(2,20)_ = 2.719, *p* = 0.090; time *F*_(5,100)_ = 4.3991, *p* = 0.001; interaction *F*_(10,100)_ = 1.391, *p* = 0.2195; Fig. 4*D*)^o^.

**Fig. 5.**
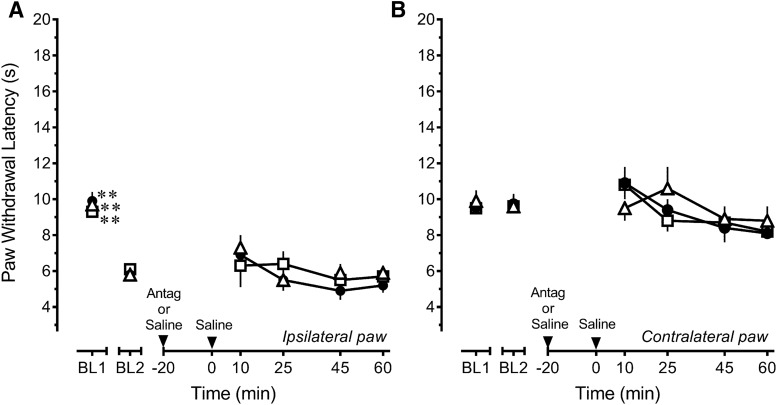
Persistent inflammatory injury does not establish tonic nicotinic cholinergic signaling in the rostral ventromedial medulla. In rats that received an intraplantar injection of CFA 2 weeks earlier, microinjection of 5 μg DHβE (☐, *n* = 6) or 1 μg MLA (▵, *n* = 6) did not alter either heat hyperalgesia in the ipsilateral, inflamed hind paw (***A***) or antinociception in the contralateral, uninflamed hind paw (***B***) compared to saline (●, *n* = 8). Data are mean ± SEM. BL1 refers to paw withdrawal latency before and BL2 refers to paw withdrawal latency after injection of CFA. **p < 0.01 compared with BL2 values. Two-way repeated-measures ANOVA followed by Holm–Sidak test.

To assess the possible development of tonic nAChR activity after inflammatory injury, the effects of the antagonists by themselves were also examined in the RVM of rats that received an intraplantar injection of CFA 2 weeks earlier (Fig. 5*A*, *B*). As for the pharmacological challenge of epibatidine, the antagonists were microinjected 20 min before injection of saline at the same site so that their effects could be assessed at the same time. Heat hyperalgesia in the ipsilateral hind paw was unaffected by either DHβE or MLA (treatment *F*_(2,17)_ = 0.287, *p =* 0.754; time *F*_(5,85)_ = 25.112, *p* < 0.001; interaction *F*_(10,85)_ = 0.576, *p* = 0.829)^p^ (Fig. 5*A*). Paw withdrawal latency in the contralateral, uninflamed hind paw was also unaffected by either antagonist (treatment *F*_(2,17)_ = 0.281, *p =* 0.758; time *F*_(5,85)_ = 3.364, *p* = 0.008; interaction *F*_(10,85)_ = 0.609, *p* = 0.802)^q^ (Fig. 5*B*). These data suggest that minimal, if any, tonic activation of α4β2 or α7 nAChRs in the RVM develops as a consequence of persistent inflammatory injury.

### Peripheral inflammatory injury does not alter the number or affinity of α4β2 nAChRs in the RVM

Initial experiments established optimal assay conditions. Association analysis demonstrated that the amount of [^3^H]epibatidine bound rapidly increased after radioligand addition, and steady-state binding was achieved after ∼2 h of incubation (data not shown). As such, a 3-h incubation time was selected for all subsequent experiments. The association constant *k_on_* was 1.056 × 10^9^ M^−1^ · min^−1^. The amount of bound [^3^H]epibatidine began to decrease immediately after dissociation was induced by the addition of the competing ligand nicotine, and specific binding became negligible after ∼11 h (data not shown). The dissociation constant *k_off_* was 6.72 × 10^−3^ per min. The binding affinity for epibatidine, $K_{d}=\frac{k_{off}}{k_{on}}$, was calculated to be 6.37 pm, consistent with values found in the literature (Houghtling et al., 1995; Whiteaker et al., 1998).

**Fig. 6.**
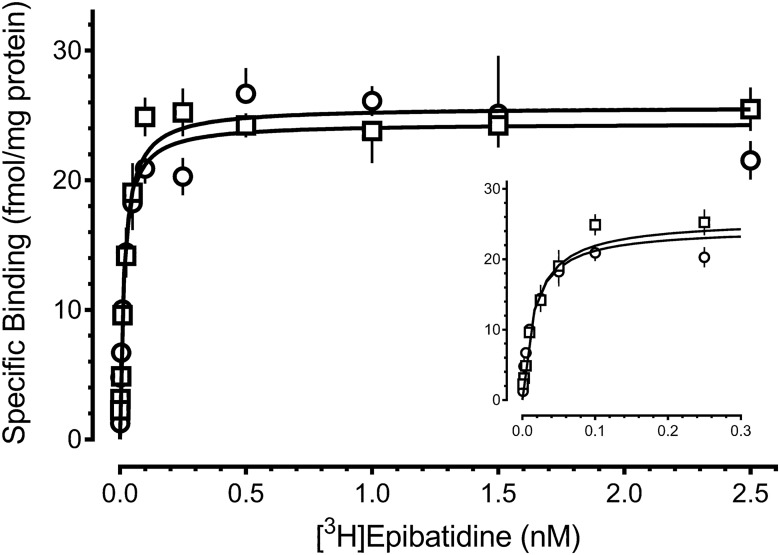
Persistent inflammatory injury does not alter the number or affinity of α4β2 nicotinic cholinergic receptors in the rostral ventromedial medulla. Saturation isotherms for binding of [^3^H]epibatidine to membrane homogenates of the rostral ventromedial medulla of rats 4 h after intraplantar injection of saline (☐) or CFA (∘) in the hind paw. Symbols represent the mean ± SEM. of triplicate samples. Results from a single representative experiment are illustrated. Specific binding was determined by subtracting nonspecific binding from total binding at each of these concentrations. Specific binding data were fitted using nonlinear regression in GraphPad Prism. Inset, Magnified view of curves at low radioligand concentrations.

Fig. 6 illustrates representative saturation isotherms for [^3^H]epibatidine binding in RVM tissue from saline- and CFA-treated rats. In saline-treated rats, the *B*_max_ values ranged from 18.9 to 23.6 fmol/mg protein, and the *K_d_* values ranged from 9.8 to 13.7 pm, in good agreement with those calculated using kinetic parameters. Comparison of saturation binding in the RVM of saline- and CFA-treated rats indicated that neither *B*_max_ (treatment *F*_(1,16)_ = 0.024, *p* = 0.878; time F_(2,16)_ = 2.050, *p* = 0.160; interaction *F*_(2,16)_ = 0.168, *p* = 0.847)^r^ nor *K_d_* (treatment F_(1,16)_ = 0.362, *p* = 0.968; time *F*_(2,16)_ = 0.002, *p* = 0.702; interaction *F*_(2,16)_ = 0.067, *p* = 0.935)^s^ changed after peripheral inflammatory injury (Table 2).

**Table 2.** Persistent inflammation does not alter the number (*B*_max_) or affinity (*K_d_*) of α4β2 nicotinic acetylcholine receptors in the rostral ventromedial medulla.

Treatment	*B*_max_, fmol/mg protein	*K_d_*, pm
4 h		
Saline	22.5 ± 2.0	12.5 ± 2.4
CFA	23.6 ± 0.5	13.7 ± 0.7
4 d		
Saline	19.4 ± 2.1	12.5 ± 4.2
CFA	20.0 ± 1.4	12.3 ± 1.6
2 weeks		
Saline	20.0 ± 1.1	11.1 ± 2.0
CFA	18.9 ± 2.6	9.8 ± 3.6

Saturation isotherms were generated for the binding of [^3^H]epibatidine in membrane homogenates prepared from rats 4 h, 4 d, or 2 weeks after intraplantar injection of saline or CFA. Values represent the mean ± SEM. of three to five independent experiments. Two-way ANOVA (treatment × time) for each binding parameter identified no statistical differences.

The thickness of the injected hind paws in the cohort of CFA-treated rats used for the binding studies was significantly greater than that of the corresponding hind paw of saline-treated rats [4 h: 9.0 ± 0.2 vs. 5.8 ± 0.1; 4 d: 9.6 ± 0.1 vs. 6.1 ± 0.1; 2 weeks: 10.3 ± 0.2 vs. 6.3 ± 0.1 mm (4 h Welch-corrected *t* = 17.12, df = 10.59; 4 d Welch-corrected *t* = 22.54, df = 22.63; 2 weeks Welch-corrected *t* = 16.44, df = 10.18, *p* < 0.001 for all three time points; *n* = 9–15 at each time point)^t^] or the uninflamed, contralateral hind paws (data not shown). The thickness of the ipsilateral hind paw of saline-treated rats did not change at any time point (data not shown).

## Discussion

This study first confirmed that the RVM is an important site of action for the antinociceptive effects of α4β2-preferring nAChR agonists (Iwamoto, 1991; Bitner et al., 1998; Curzon et al., 1998) and characterized the receptor through which epibatidine acts in uninjured rats. Subsequent experiments characterized the ramifications of persistent inflammatory pain on the actions of epibatidine. Epibatidine retained its ability to alleviate heat hyperalgesia as long as 2 weeks after injury. Although mediated by α4β2 nAChRs 4 h after injury, by 2 weeks the antihyperalgesia was no longer substantially mediated by these receptors. Its antinociceptive effect, determined in the contralateral hind paw, was abolished in a time-dependent manner. Moreover, in contrast to naive rats, the antinociceptive effect was no longer mediated by α4β2 nAChRs as early as 4 h after CFA. These changes occurred in the absence of changes in the *B*_max_ or *K_d_* of α4β2 nAChRs in the RVM.

### Pharmacology of the antinociceptive effect of epibatidine in the RVM of uninjured rats

In uninjured rats, microinjection of epibatidine in the RVM dose-dependently increased PWL. As reported (Curzon et al., 1998), the slope of the dose–response curve was relatively shallow and may reflect rapid desensitization of α4β2 nAChRs (Zhang et al., 2012). Also, the first measure of PWL was not made until 10 min after microinjection to minimize any effects of handling. Thus, both the potency and efficacy of epibatidine may be underestimated here.

In terms of affinity and potency, epibatidine is ∼300-fold selective for α4β2 over α7 nAChRs (Wonnacott and Barik, 2007). It was used as a pharmacological probe in this study because it is several orders of magnitude more potent than nicotine, which is less selective for α4β2 over α7 nAChRs (Wonnacott and Barik, 2007). The ability of DHβE to completely block effects of epibatidine in the RVM reaffirms (Curzon et al., 1998) that the principal mechanism of action of epibatidine in the RVM entails activation of heteromeric nAChRs, principally α4β2. This subtype accounts for 70%–90% of [^3^H]nicotine binding sites in the brain (Daly, 2005; Wonnacott and Barik, 2007; Marks et al., 2010). However, in mouse brain, one-third of the low-affinity epibatidine binding sites are attributed to α7 nAChRs (Marks et al., 2010). The effects of epibatidine were therefore also challenged with the α7 nAChR antagonist, MLA. Interestingly, MLA partially attenuated the effect of epibatidine. Autoradiography has detected only very low levels of α7 nAChRs in the RVM (Mugnaini et al., 2002; Tribollet et al., 2004; Maier et al., 2011; but see Clarke et al., 1985). Thus, α7 nAChRs may make a minor contribution to epibatidine-induced antinociception in the RVM in the uninjured state. Given that α7 nAChRs are expressed in several pain modulatory nuclei that project to the RVM, such as the periaqueductal gray and amygdala (Clarke et al., 1985), systemically administered α7 nAChR agonists may be able to indirectly engage the neural circuitry of the RVM.

MLA also has nanomolar affinity for α6β2 nAChRs, albeit about 30-fold less than for α7 nAChRs (Mogg et al., 2002; Wonnacott and Barik, 2007). The ability of MLA to partially attenuate the effects of epibatidine may therefore also reflect a contribution of α6-containing nAChRs such as α3/α6β2β3. The distribution of α6-containing nAChRs in the CNS is highly restricted (Picciotto et al., 2001), and the majority of studies have focused on their role in the presynaptic facilitation of dopamine release in the reward pathways (Yang et al., 2009). However, α6-containing nAChRs have been implicated in nicotine-induced release of norepinephrine in the hippocampus (Azam et al., 2010), and high levels of α6 mRNA are present in the locus coeruleus (Le Novère et al., 1996; Léna et al., 1999). Noradrenergic afferents to the RVM modulate the activity of bulbospinal pain inhibitory and facilitatory neurons in the RVM (Hammond et al., 1980; Sagen and Proudfit, 1985; Haws et al., 1990; Bie et al., 2003). Thus, it is possible that a component of epibatidine’s effects could be mediated by actions at α6-containing nAChRs (Champtiaux et al., 2002) situated on noradrenergic terminals in the RVM.

### Inflammatory injury changes the mechanism by which epibatidine alleviates hyperalgesia and diminishes its antinociceptive efficacy

Numerous studies have reported that agonists for α4β2, α7, or α3/α6β2β3 nAChRs alleviate hyperalgesia when systemically administered in a number of inflammatory models including CFA, formalin, or carrageenan (Kesingland et al., 2000; Medhurst et al., 2008; Feuerbach et al., 2009; Gao et al., 2010; Zhu et al., 2011; AlSharari et al., 2012; Munro et al., 2012; Nirogi et al., 2013). Much less is known about the actions of nAChR agonists selective for receptors containing α5 and α6 subunits, in part because of the absence of selective full agonists. One study has correlated the antinociceptive potency of α4β2 nAChR agonists in the formalin test to their potency to desensitize this receptor, particularly when it contains an α5 subunit (Zhang et al., 2012), and another demonstrated that the anti-allodynic effect of nicotine in the formalin test was dependent on the presence of an α5 subunit (Bagdas et al., 2015). With respect to the α6 subunit, deletion of this subunit greatly diminished the antihyperalgesic effect of nicotine in CFA-treated mice (Wieskopf et al., 2015).

Studies of the antihyperalgesic effects of nAChR agonists have rarely characterized efficacy or potency as a function of time after injury or probed pharmacological specificity any later than 4 d after injury, nor have they investigated actions in supraspinal pain modulatory circuitry. This study demonstrated that epibatidine retained its antihyperalgesic effects for as long as 2 weeks after inflammatory injury. In the period immediate to injury, its antihyperalgesic effect was completely antagonized by DHβE, consistent with activation of α4β2 nAChRs. However, when reassessed 2 weeks later, epibatidine’s antihyperalgesic effect was only partially antagonized by DHβE. Of note, MLA did not antagonize the antihyperalgesic effects of epibatidine at either time. The present findings point to a striking change in the mechanism by which epibatidine alleviates hyperalgesia to one that does not involve α7 or α3/α6β2β3, and only minimally involves α4β2 nAChRs, in a key pain modulatory pathway.

In contrast to its antihyperalgesic effects, epibatidine’s antinociceptive efficacy progressively declined such that it was without effect by 2 weeks. These findings are consistent with the proposal that chronic pain conditions lead to a loss of the analgesic efficacy of nicotine. Moreover, as early as 4 h of injury, the antinociceptive effect of epibatidine was no longer mediated by α4β2 or α7 nAChRs. Whether this is due to changes in subunit composition, such as a downregulation of α5 subunits that assemble with α4β2 subunits, remains to be determined.

### Inflammatory injury and α4β2 nAChRs in the RVM

The few studies that have assessed how persistent pain states alter the expression of nAChRs in the CNS confined their analysis to peripheral nerve injury. Spinal nerve ligation did not alter the *B*_max_ or *K_d_* of epibatidine binding sites in the dorsal horn, but an increase in the ability of nicotine to displace epibatidine suggested a change in receptor subtype (Young et al., 2008). Partial ligation of the sciatic nerve increased binding to α4β2 nAChRs in the thalamus (Ueda et al., 2010). However, it did not change epibatidine binding in the RVM, as was also observed here with CFA. Chronic constriction injury decreased β4 subunits in hippocampus, thalamus, and spinal cord (Xanthos et al., 2015).

In contrast, inflammatory injury did not change the affinity or number of α4β2 nAChRs in the RVM. A decrease in the number or affinity of α4β2 nAChRs therefore does not appear to be responsible for the loss of antinociceptive efficacy or contribute to the change in receptors that mediate its antihyperalgesic effects. However, several caveats should be noted. Changes in receptor number or subunit composition limited to subpopulations of RVM neurons may not have been detected by radioligand binding in tissue homogenates. An example would be a downregulation of α5 subunits, which can affiliate with α4β2 nAChRs and contribute to the antinociceptive effects of nicotine (Ramirez-Latorre et al., 1996; Jackson et al., 2010; but see Xanthos et al., 2015). Finally, evidence is accruing for an interaction of nAChRs and G-proteins (Kabbani et al., 2013) such that alterations in subcellular signaling pathways downstream of the receptor may play a role.

### Effect of inflammatory injury on pain modulatory pathways and implications for nicotinic modulation of nociception

Inflammatory injury may alter the affinity, number, and subtypes of nAChRs through which agonists or endogenously release acetylcholine act to modulate nociception. However, it is also possible that the antihyperalgesic and antinociceptive effects of nAChR agonists are altered independently of changes in the nAChR, as occurred here in the RVM. For example, presynaptic nAChRs promote neurotransmitter release (Picciotto et al., 2012). The antinociceptive and antihyperalgesic effects of nAChR agonists may therefore be altered secondary to injury-induced plasticity in other neurotransmitter systems whose release nAChR agonists promote, e.g., a reduction in terminal content of the neurotransmitter or a downregulation of its corresponding receptor. For example, persistent inflammatory injury enhances the antihyperalgesic or antinociceptive actions of opioid, α-amino-3-hydroxy-5-methyl-4-isoxazolepropionic acid (AMPA), and *N*-methyl-d-aspartate receptor agonists in the RVM (Hurley and Hammond, 2000; Guan et al., 2002, 2003, 2004). A facilitation of opioid peptide or glutamate release in the RVM would be inconsistent with the loss of antinociceptive efficacy of epibatidine. However, prior activation of α4β2 nAChRs in synaptosomes of the nucleus trigeminal caudalis is reported to increase internalization and functionally downregulate AMPA receptors with a concomitant decrease in excitatory amino acid release (Samengo et al., 2015). If operative in the RVM, an epibatidine-induced decrease in functional AMPA receptors could contribute to a decrement in antinociception. Peripheral stimulation during chronic inflammatory pain states is also associated with release of two pronociceptive neurotransmitters in the RVM: GABA (Gilbert and Franklin, 2001) and substance P (Hamity et al., 2014). Epibatidine-induced augmentation of GABA or substance P release may also contribute to the loss of antinociception. The development of analgesics based on activation of nAChRs for the relief of persistent pain will require a better understanding of how peripheral injury affects the expression of different nAChR subtypes in specific sites, as well how it alters the activity and function of the neurotransmitter systems that these receptors engage.

### Potential ramifications for development of positive allosteric modulators as analgesics

Positive allosteric modulators (PAMs) for α4β2 and α7 nAChRs are a promising approach to potentiate endogenously released acetylcholine or low doses of orthosteric agonists and thereby circumvent the narrow therapeutic index of orthosteric agonists (Lee et al., 2011; Nirogi et al., 2013). Both α4β2 and α7 nAChR PAMs are efficacious in certain preclinical pain models (Zhu et al., 2011; Freitas et al., 2013; Bagdas et al., 2016; but see Gao et al., 2010). When administered alone, the efficacy of PAMs is dependent on release of endogenous acetylcholine. In this study, microinjection of DHβE or MLA did not alter PWL in either uninjured rats or CFA-treated rats. These data suggest that there is little to no tonic release of acetylcholine in the RVM that would engage α4β2 or α7 nAChRs and that the RVM would be an unlikely site of action for the analgesic effects of systemically administered PAMs.

### Conclusion

A loss of nAChR agonist efficacy has not been modeled in the preclinical literature to date, and no studies have compared antinociceptive efficacy between uninjured and persistent pain states. At first glance, the loss of antinociceptive efficacy in the face of the retention of antihyperalgesic efficacy appears puzzling. The former is compatible with the hypothesis that persistent pain leads to a loss of nicotine’s analgesic efficacy, but the latter is not. Several possibilities merit consideration and indicate potential avenues of future investigation. First, although the antihyperalgesic efficacy of epibatidine was retained in these experiments, it is not known whether this is the clinical experience. The extant literature principally concerns nicotine’s effects in the acute and postoperative pain setting. Indeed, the continued presence of chronic pain in smokers and the positive correlation between severity of pain and smoking would suggest that it is not (Bakhshaie et al., 2016). Second, reward and pain modulatory pathways are intimately linked (Navratilova and Porreca, 2014). It remains to be determined whether persistent pain also alters the expression of nAChRs in reward pathways or diminishes the rewarding effects of nicotine, which could drive those with chronic pain to smoke more. Finally, these differential effects also suggest that the RVM circuitry that mediates antihyperalgesia and antinociception can be dissociated. In summary, the present findings are the first evidence in a preclinical model of persistent pain of adaptive changes in a critical nucleus that result in an eventual loss of antinociceptive efficacy of a nAChR agonist and a change in the mechanism by which it produces antihyperalgesia. The exact nature of these changes, how they affect the actions of nicotine and other more subtype selective nAChR agonists in the RVM, as well as systemically administered nicotine, are the focus of ongoing investigation.

## Synthesis

Reviewing Editor: Mary Heinricher, Oregon Health & Science University Decisions are customarily a result of the Reviewing Editor and the peer reviewers coming together and discussing their recommendations until a consensus is reached. When revisions are invited, a fact-based synthesis statement explaining their decision and outlining what is needed to prepare a revision will be listed below. The following reviewer(s) agreed to reveal their identity: Michael Morgan, Bridget Lumb.

The experience of pain is dynamic, in part because of time-dependent changes in the influence of pain-modulating systems as a long-lasting pain state develops and is maintained. This manuscript addresses adaptive changes in nicotinic receptor pharmacology in the rostral ventromedial medulla (RVM) of rats in a persistent inflammatory pain state. The RVM is the output node of a principal brain pain-modulating circuit.

In a series of thorough and well-documented behavioral studies, the authors found that the antinociceptive (”analgesic”) effect of a nicotinic agonist (epibatadine) applied directly in the RVM is reduced over a period of weeks. In normal animals, the analgesic effect is mediated by alpha4beta2 and alpha7 receptors. Within a few hours after inflammation is triggered, these receptors are no longer required (although epibatadine still has an analgesic effect at this time). In addition, epibatadine has an anti-hyperalgesic effect, reversing hyperalgesia in the inflamed paw, and this anti-hyperalgesia epibatadine is maintained for at least two weeks. Again, the underlying pharmacology changes, such that the alpha4beta2 antagonist has a lesser effect, and the alpha7 receptor antagonist is without effect on epibatadine-induced anti-hyperalgesia. Ligand binding studies suggest that these functional changes cannot easily be accounted for by changes in receptor binding or number.

The focus of the report is the behavioral pharmacological analysis, and these are extraordinarily well done, with comparison of the inflamed paw (where anti-hyperalgesia is measured) and the contralateral, uninflamed paw (where analgesia can be tested). Appropriate controls, including blinding the experimenter to the drug treatment, are in place. The authors also acknowledge important limitations (e.g., that tissue homogenates might not have sufficient sensitivity to reveal changes in receptor number).

The issue of a potential impact of inflammation on ligand binding would benefit from a more thoughtful discussion. The authors state that peripheral stimulation was not employed in the binding studies because “...nociceptive testing can alter receptor expression independently of the inflammatory injury.” However, for this very reason, the behavioral data and the ligand binding data are not directly comparable.

Further discussion of other (nicotinic?) receptors in inflamed animals would help the reader put these data into a larger context. That is, the maintained anti-hyperalgesic action of epibatadine despite the fact that presumably relevant receptor antagonists no longer block that effect is a key finding, and the authors state at one point that the effects of epibatadine may not be mediated by nicotinic receptors. However, further discussion (pp. 25-26) seems to assume that endogenous acetylcholine (or epibatadine) is acting on nicotinic receptors to produce circuit-level changes, or even plasticity in relevant circuits.

Finally, the idea that loss of antinociceptive efficacy with maintained anti-hyperalgesic efficacy would lead to increased smoking in individuals with chronic pain is puzzling since the net behavioral (antihyperalgesic) effect of epibatadine is maintained.

Additional suggestions for improving clarity and impact:

The abstract would be enhanced by some background information to provide a clearer rationale/hypothesis.

Although there is a rationale for focusing on the alpha2 antagonist, that for trying the alpha7 antagonist was never clearly articulated.

Methods. What is meant by “...microinjection sites outside this region were not included in the pharmacological analysis.” This is important as they are included in Fig. 2 and serve as controls.

Why are measurements of the degree of inflammation given for binding studies alone? To draw a clear parallel between the behavioral studies and the binding experiments, it would be useful to see that the degree of inflammation was comparable.

Statistical analysis. On p. 14, description of Fig. 1B. Shouldn't the error term have 64 df, rather than 62? Please verify df and other analysis details throughout. Why was Welch's t-test used if, as stated in the methods, groups had equal variances? Throughout the manuscript the authors refer to “complete” and “marginal” antagonism of epibatadine's behavioral effects. This seems to be based on the resulting p-values. P-values do not represent the size of an effect, and this should be revisited.

Figure 3 caption. It would help to have n's as in the other figures.

Fig. 1. The symbols in the inserted D/R graph should be replaced with the appropriate symbol from Fig. 1A. This would provide a visual link between the two graphs and an in-text legend for 1A. In addition, the x-axis on the inserted graph would be easier without the 0.5 and 5 doses listed.

Fig. 4. The caption for Fig. 4D should state that these rats received epibatadine as is done when describing the other graphs in Fig. 4.
